# Real-world data on immune checkpoint inhibitors in advanced sarcomas across multiple European institutions

**DOI:** 10.2340/1651-226X.2025.43135

**Published:** 2025-06-10

**Authors:** Stefania Kokkali, Ana Dolcan, Kjetil Boye, Anastasios Kyriazoglou, Ioannis Boukovinas, Foteini Kalofonou, Anna Koumarianou, Natalia Asimakopoulou, Eleftherios Vorrias, Konstantinos Tsapakidis, Eleni Georgaki, Anna Boulouta, Leonidas Mavroeidis, Magnus Harneshaug, Stamatios Theocharis, Robin L. Jones, Antonia Digklia

**Affiliations:** a Oncology Unit, Second Department of Medicine, University of Athens, Hippocratio General Hospital of Athens, Athens, Greece; b Department of Oncology, Lausanne University Hospital, Lausanne, Switzerland; c Department of Oncology, Oslo University Hospital, Oslo, Norway; d Second Department of Internal Medicine, Oncology Unit, Attikon University Hospital, Athens, Greece; e Bioclinic Thessaloniki Medical Oncology Unit, Thessaloniki, Greece; f The Royal Marsden NHS Foundation Trust and Institute of Cancer Research, London, UK; g Fourth Department of Internal Medicine, Attikon University Hospital, Athens, Greece; h First Department of Medical Oncology, Metropolitan General Hospital, Athens, Greece; i Department of Medical Oncology, General University Hospital of Heraklion, Heraklion, Greece; j Department of Medical Oncology, University General Hospital of Larissa, Larissa, Greece; k First Department of Pathology, Medical School, National and Kapodistrian University of Athens, Athens, Greece

**Keywords:** Immunotherapy, immune checkpoint inhibitor, programmed death-1 (PD-1), programmed death-ligand 1 (PD-L1), sarcomas

## Abstract

**Background:**

Following the success of immune checkpoint inhibitors (ICI) in other cancer types, their role is being evaluated in sarcomas. They have been assessed as monotherapy, or in combination with other ICI, chemotherapeutic drugs and tyrosine kinase inhibitors (TKI) in several clinical trials. So far the results have been limited to non-selected sarcoma populations. Further work is required to select patients who will benefit from immunotherapy.

**Patients and methods:**

We conducted a pooled retrospective analysis of the use of ICI in patients with advanced sarcomas in multiple European institutions. ICI-based treatments included ICI monotherapy (*n* = 43, 59.7%), double ICI (*n* = 5, 6.9%), ICI plus TKI (*n* = 21, 29.2%) and ICI plus chemotherapy (*n* = 3, 4.2%).

**Results:**

Seventy-two patients from 10 European institutions, with metastatic (87.5%) or locally advanced (12.5%) disease were included. The most common subtype was undifferentiated pleomorphic sarcoma (16.7%), followed by leiomyosarcoma (12%), liposarcoma (10%) and angiosarcoma (9.7%). The median number of prior lines of systemic therapy was 2 (0–8). The objective response rate was 34.4% and was higher in combination regimens versus ICI monotherapy. With a median follow-up of 20.7 months, median progression-free survival (PFS) was 4.6 and median overall survival (OS) 18.8 months. Line of therapy (1st/2nd vs. ≥ 3rd line) and best response to ICI was significantly associated with PFS and OS. Histological subtype was significantly associated with OS. Toxicity was in general manageable; only six (8.3%) patients discontinued therapy for AE.

**Interpretation:**

Our study provided additional real-world data on the outcome of ICI in patients with advanced sarcomas.

## Introduction

Redirecting the patient’s immune system, the so-called immunotherapy, has resulted in remarkable tumor responses and survival prolongation in various cancers, such as melanoma [1], non-small cell lung cancer [2] and renal cell carcinoma [3]. Following the advancement of immune checkpoint inhibitors (ICI) in other cancers, there is heightened interest in exploring their role in sarcomas. They have been investigated in small phase 2 clinical trials including various sarcoma subtypes. The first trials to use ICI in sarcomas either as monotherapy with programmed death-1 (PD-1) or programmed death-ligand 1 (PD-L1) inhibitors [4, 5], or in combination with CTLA-4 inhibitors [4, 6], demonstrated limited efficacy in unselected sarcoma population with some isolated radiological responses. Subsequently, ICI combinations with tyrosine kinase inhibitors (TKI) or chemotherapy were evaluated in phase 2 clinical trials in a variety of sarcoma subtypes, with more promising results [7, 8].

All studies of ICI in soft tissue sarcomas (STS) have demonstrated clinical activity in alveolar soft part sarcoma (ASPS) [4, 7, 8], resulting in the approval of atezolizumab by the FDA for this subtype in December 2022. Their efficacy in other STS histotypes is less clear, with some observed responses in patients with undifferentiated pleomorphic sarcoma (UPS), dedifferentiated liposarcoma (DDLPS), myxofibrosarcoma (MFS), leiomyosarcoma (LMS), cutaneous angiosarcoma (AS) etc. It has become clear in sarcoma research that future studies should be histotype-specific, given the heterogeneity of the disease and the need for histotype-tailored approaches. However, there are only a small number of histotype-specific trials investigating the role of immunotherapy, which include small numbers of patients. Among them, a phase 2 trial in ASPS [9], a phase 2 trial in AS [10], a phase 1/2 trial in liposarcoma (LPS) and LMS [11] and a phase 1 trial in UPS and LMS investigated the safety and efficacy of PD-1 inhibitors alone or in combination with other agents.

Therefore, the role of immunotherapy in sarcomas is still being explored and optimal strategies are yet to be defined. The understanding of molecular and immunological mechanisms underpinning sarcomas will help move the field forward. To date, there are no established biomarkers to predict the response to ICI in sarcoma patients, with some data supporting a role for the presence of tertiary lymphoid structures (TLS), defined as organized lymphoid structures containing B lymphocytes admixed to CD23+ follicular dendritic cells [12]. Analysis of the available clinical data, as well as correlative studies in clinical trials, will guide a better patient selection.

In an effort to leverage real-world data on the use of ICI, we report the results of a pooled retrospective analysis in patients with unselected advanced sarcomas treated in multiple European institutions.

## Methods

### Study design

This was a multicenter, retrospective study that included patients with advanced sarcomas treated with off-label ICI. The study was conducted by hospital-based oncologists specializing in sarcoma care and practicing in 10 European centers in Greece, Norway, Switzerland and the United Kingdom.

All consecutive patients ≥ 16 years old with a histologically confirmed diagnosis of STS or bone sarcomas (locally advanced or metastatic), who were treated in the participating centers with ICI over the last 6 years, starting between May 2018 and June 2024, were included in the study. Epithelioid sarcoma (ES) patients from the Royal Marsden Hospital were excluded from this study, as they have been included in a separate analysis. Patients were selected according to each treating physician’s discretion depending on the patient’s conditions, histotype, immunotherapy biomarkers and previous therapies, as well as institutional common practice.

We analyzed the histopathological and clinical data, as well as ICI treatment outcomes and toxicity profile. Variables of interest were demographics, histological subtype, biomarkers of immunotherapy, localization of metastases, surgery of the primary tumor and perioperative chemotherapy regimen, adverse events (AE) to the ICI regimen according to the NCI Common Terminology Criteria for Adverse Events (CTCAE) version 5.0, prior therapies for systemic disease and outcome with ICI. Patients were monitored for AE at every follow-up assessment and whenever clinically indicated.

This study was designed and conducted in accordance with the ethical principles laid down in the Declaration of Helsinki and all applicable local rules and regulations. It was approved by the Institutional Review Board of each participating institute.

### Endpoint definitions

Tumor response to the ICI regimen was evaluated by the participating physicians according to local and institutional common practice and Response Evaluation Criteria in Solid Tumors (RECIST) v1.1. Progression-free survival (PFS) was defined as the time from the date of treatment initiation until the first radiographic documentation of objective tumor progression or death regardless of cause. Overall survival (OS) was defined as the interval between treatment initiation and the date of death regardless of the cause or the date of the last follow-up. Objective response rate (ORR) was defined as the proportion of patients with either a radiological complete response (CR) or a partial response (PR) as best response by RECIST v1.1.

### Treatments

Immunotherapy regimens included ICI monotherapy or ICI combination with TKI, other ICI or chemotherapy. Nivolumab +/- ipilimumab, pembrolizumab and atezolizumab were administered at standard doses used in other tumor types. ICI in combination with TKI was given according to phase 2 clinical trial dosing regimens [7, 8]. Finally, an ICI was combined with chemotherapeutic drug at standard doses.

There were no predefined limits to the number of ICI cycles administered and treatment continued until unacceptable toxicity or as long as the treating physician judged there was clinical benefit.

### Statistical analysis methods

Descriptive statistics were used to summarize patient, disease and treatment characteristics (median, minimum and maximum for continuous variables; numbers and percentages for categorical variables). Comparisons between categorical variables were conducted using chi-square test. PFS and OS, along with the respective 95% CIs were estimated according to the Kaplan–Meier method. For OS analysis, patients who were alive were censored at their last follow-up date. The association of patient and disease characteristics of interest with PFS and OS was examined using the log-rank test. Statistical analyses were conducted using the SPSS 28 (IBM Corp., Armonk, NY) statistical analysis software. Significance level was defined at 0.05.

## Results

### Patients’ characteristics

Seventy-two patients were included in this analysis. Their demographics and complete clinical characteristics are outlined in Table 1. Median age at ICI initiation was 56 (range, 16–94) years and the majority of patients were male (*n* = 44, 61.1%). Almost 90% of the patients had metastatic disease. There were 69 cases of STS and three cases of bone sarcomas (two chordomas and one dedifferentiated chondrosarcoma [CS]). UPS (16.7%) and LMS (16.7%) were the most prevalent subtypes, followed by LPS (13.9%) and AS (9.7%). The primary tumor was located in the extremities (31.9%), in the trunk (25%), in the retroperitoneum (19.4%), in the head and neck (13.9%), in the uterus (8.3%) and in the spermatic cord (1.4%). More than half of the patients had undergone surgery of the primary tumor and approximately a quarter of the patients had received perioperative chemotherapy.

**Table 1:** Patient characteristics (N = 72).

Characteristic	*N* (%)
**Sex**	
Male	44 (61.1)
Female	28 (38.9)
**Age at ICI initiation (years), median (range)**	56 (16–94)
**Stage**	
Metastatic	63 (87.5)
Locally advanced	9 (12.5)
**Histological subtype**	
UPS	12 (16.7)
LMS/of which uLMS	12 (16.7) /5 (6.9)
LPS/of which DDLPS	10 (13.9) / 7 (9.7)
AS	7 (9.7)
Kaposi sarcoma	4 (5.6)
Undifferentiated sarcoma	3 (4.2)
ASPS	3 (4.2)
MFS	3 (4.2)
Other^1^	18 (25.0)
**Site of primary tumor**	
Extremities	23 (31.9)
Trunk	18 (25.0)
Retroperitoneum	14 (19.4)
Head and neck	10 (13.9)
Uterus	6 (8.3)
Spermatic cord	1 (1.4)
**Metastatic sites**	
Lung	39 (54.2)
Liver	11 (15.3)
Other	41 (56.9)
**Number of metastatic sites, median (range)**	2 (1–4)
**Adjuvant/neoadjuvant chemotherapy**	23 (31.9)
**Surgery of the primary tumor**	57 (79.2)
**Number of prior lines of systemic therapy**	2 (0–8)
**Prior systemic therapies**	
Anthracycline-based	45 (62.5)
Gemcitabine-based	22 (30.6)
Trabectedin	18 (25.0)
TKI	18 (25.0)
Other	37 (51.4)

1 Other histological subtypes: two cases of chordoma, epithelioid sarcoma, fibrosarcoma, pleomorphic dermal sarcoma, rhabdomyosarcoma and one case of clear cell sarcoma-like tumor of the gastrointestinal tract, dedifferentiated chondrosarcoma, desmoplastic small round cell tumor, fibromyxoid sarcoma, intimal sarcoma of the heart, malignant granular cell tumor, malignant peripheral nerve sheath tumor, synovial sarcoma.

AS: angiosarcoma; ASPS: alveolar soft part sarcoma; DDLPS: dedifferentiated liposarcoma; ICI: immune checkpoint inhibitor; LMS: leiomyosarcoma; LPS: liposarcoma; MFS: myxofibrosarcoma; TKI: tyrosine kinase inhibitor; uLMS: uterine leiomyosarcoma; UPS: undifferentiated pleomorphic sarcoma.

Patients had received a median number of two prior lines of systemic therapy (Table 1). Immunotherapy regimens consisted of ICI monotherapy in 43 (59.7%) cases (pembrolizumab: *n* = 27, nivolumab: *n* = 15), combination of ICI with TKI in 21 (29.2%) cases (pembrolizumab/axitinib: *n* = 12, nivolumab/cabozantinib: *n* = 7, nivolumab/sunitinib: *n* = 1, atezolizumab/pazopanib: *n* = 1), double ICI in five (6.9%) cases (nivolumab/ipilimumab) and combination with chemotherapy in three (4.2%) cases (atezolizumab/eribulin, nivolumab/ifosfamide, nivolumab/gemcitabine/docetaxel).

### Clinical efficacy

At the time of the analysis, 15 patients (20.8%) were still on treatment with ICI, whereas the remaining 57 had discontinued therapy (due to progressive disease [PD] in 43 [59.7%], toxicity in seven [9.7%] [of which six were for immune-related AE and one was for secondary to anthracycline acute myeloid leukemia], death in four [5.6%], treatment completion in two [2.8%] and patient’s preference in one [1.4%]). Among the 64 assessable patients, there were three (4.7%) CRs and 19 (29.7%) PRs as best response to ICI, translating into an ORR of 34.4%. Table 2 shows the ORR according to sarcoma subtype. Eighteen patients (28.1%) exhibited stable disease as the best response and 25 (39.1%) PD.

**Table 2:** Objective response rate according to sarcoma histotype.

Histological subtype	*N*	CR	PR	Response rate
		*N* (%)	*N* (%)	%
UPS	12	2 (16.7)	5 (41.7)	58.3
LMS/uLMS	12/5	0	4 (33.3)/1 (20)	33.3/20.0
LPS/DDLPS	10/7	0	3 (30)/3 (42.9)	30.0/42.9
AS	7	0	2 (28.6)	28.6
Kaposi sarcoma	4	0	1 (25)	25.0
Undifferentiated sarcoma	3	1 (33.3)	0	33.3
ASPS	3	0	1 (33.3)	33.3
Pleomorphic dermal sarcoma	2	0	1 (50.0)	50.0
Dedifferentiated Chondrosarcoma	1	0	1 (100)	100
MFS	3	0	1 (33.3)	33.3

AS: angiosarcoma; ASPS: alveolar soft part sarcoma; CR: complete response; DDLPS: dedifferentiated liposarcoma; LMS: leiomyosarcoma; LPS: liposarcoma; MFS: myxofibrosarcoma; PR: partial response; uLMS: uterine leiomyosarcoma; UPS: undifferentiated pleomorphic sarcoma.

For 24 patients there were available biomarkers for immunotherapy (PD-L1, microsatellite instability [MSI] and tumor mutational burden [TMB]). Only two tumors were MSI-high, 11 were positive for PD-L1 (≥ 1%) and six had TMB >10 mutations/Mb in 17 patients in total (two of them were positive for two immunotherapy biomarkers). Table 3 shows the status of biomarkers for the 22 patients who achieved an objective response to ICI therapy. Biomarker data were available for eight responders and five of them were positive for at least one biomarker.

**Table 3:** Biomarker status in responders to ICI therapy.

No.r	Best response	PD-L1 value	TMB value	MSI	Histological subtype
1	CR	N/A	N/A	High	Undifferentiated sarcoma
2	CR	Ν/Α	Ν/Α	Ν/Α	UPS
3	CR	N/A	N/A	N/A	UPS
4	PR	N/A	N/A	N/A	DDLPS
5	PR	N/A	N/A	N/A	UPS
6	PR	N/A	N/A	N/A	AS
7	PR	20%	N/A	N/A	UPS
8	PR	N/A	N/A	N/A	LMS
9	PR	N/A	8.9 mut/Mb	N/A	AS
10	PR	>95%	38.8 mut/Mb	N/A	Pleomorphic dermal sarcoma
11	PR	NA	N/A	N/A	DDLPS
12	PR	NA	1.4 mut/Mb	N/A	Dedifferentiated chondrosarcoma
13	PR	N/A	N/A	N/A	UPS
14	PR	TC < 1%, IC = 1%	13.4 mut/Mb	low	DDLPS
15	PR	N/A	0	low	ASPS
16	PR	N/A	N/A	N/A	MFS
17	PR	N/A	N/A	high	LMS
18	PR	N/A	N/A	N/A	UPS
19	PR	N/A	N/A	N/A	LMS
20	PR	N/A	N/A	N/A	UPS
21	PR	N/A	N/A	N/A	Kaposi sarcoma
22	PR	0%	N/A	low	LMS

AS: angiosarcoma; ASPS: alveolar soft part sarcoma; CR: complete response; DDLPS: dedifferentiated liposarcoma; IC: immune cells; LMS: leiomyosarcoma; MFS: myxofibrosarcoma; MSI: microsatellite instability; N/A: not available; PD-L1: programmed death-ligand 1; PR: partial response; TC: tumor cells; TMB: tumor mutational burden; UPS: undifferentiated pleomorphic sarcoma.

ORR was also analyzed according to ICI regimen, as depicted in Table 4. ORR was higher (40%) with double ICI (ipilimumab/nivolumab), followed by ICI+TKI combination (33.3%) and ICI monotherapy (27.3%). However, the difference in ORR was not statistically significant (*p* = 0.64).

**Table 4:** Best response according to immune checkpoint inhibitor regimen.

	Best response to ICI – *N* (%)							
	CR	PR	ORR	SD	PD	N/A	Evaluable	Total
**ICI monotherapy**	2 (4.9)	10 (24.4)	12 (29.3)	10 (24.4)	19 (46.3)	2 (4.7)	41	43
**Double ICI**	0	2 (40)	2 (40)	2 (40)	1 (20)	0	5	5
**TKI combo**	1 (6.7)	6 (40)	7 (46.7)	5 (33.3)	3 (20)	6 (28.6)	15	21
**Chemo combo**	0	1 (33.3)	1 (33.3)	2 (66.7)	0	0	3	3
**Total (%)**	3 (4.7)	19 (29.7)	22 (34.4)	18 (28.1)	25 (39.1)	8 (12.5)	64	72

CR: complete response; combo: combination; ICI: immune checkpoint inhibitor; N/A: not available, ORR: objective response rate; PD: progressive disease; PR: partial response; SD: stable disease; TKI: tyrosine kinase inhibitor.

**Figure 1:**
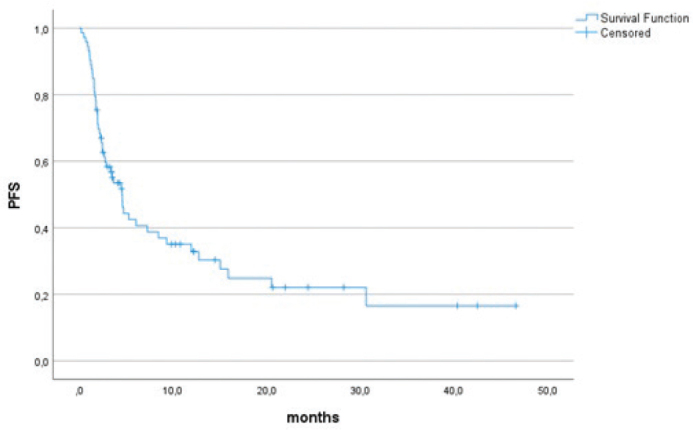
Progression-free survival of the whole cohort.

**Figure 2:**
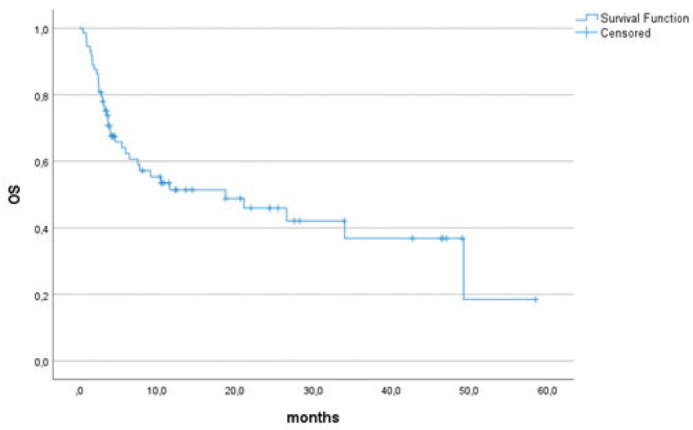
Overall survival of the whole cohort.

With a median follow-up time of 20.7 months (95% CI 9.2–32.1 months) from the initiation of therapy, 46 (63.9%) patients had PD and 37 (51.4%) had died, with a median PFS of 4.6 months (95% CI 3.2–5.9 months) (Figure 1). Median OS was 18.8 months (95% CI 1.9–35.6 months) (Figure 2).

### Subgroup analysis

We performed subgroup analysis to assess the effect of the following variables on PFS and OS: sex, stage of sarcoma, number of metastatic sites, location of the primary tumor (extremities vs. trunk vs. retroperitoneum vs. head and neck vs. uterus vs. other), histology, immunotherapy regimen (ICI monotherapy vs. double ICI vs. combination of ICI with TKI vs. combination with chemotherapy), line of treatment (ICI administered as 1st/2nd vs. ≥ 3rd line), best response to ICI and occurrence of AE. Only line of therapy (median PFS 8.5 months in the 1st/2nd line vs. 2.9 months in the ≥ 3rd line, *p* = 0.027) and best response to ICI (median PFS 30.6 months in responders vs. 2.5 months in non-responders, *p* < 0.001) had a statistically significant impact on PFS (Supplementary Figures 1, 2). In addition, we observed a longer PFS in patients with primary tumors located in the head and neck compared to other locations (median PFS 15.9 months for head and neck sarcomas vs. 4.5 months for others, *p* = 0.110), and in patients who experienced AE (median PFS 15.9 months in patients with AE vs. 2.9 months in those without AE, *p* = 0.464), notwithstanding the lack of statistical significance.

Similarly, time of therapy (median OS 49.2 months in the 1st/2nd line vs. 7.8 months in the ≥ 3rd line, *p* = 0.013), best response to ICI (median OS not reached in responders vs. 6.4 months in non-responders, *p* = 0.002) and subtype (*p* = 0.002) had a statistically significant impact on OS (Supplementary Figures 3, 4). Among the most common subtypes, the longest OS was observed in patients with LPS (median OS 49.2 months), followed by LMS (median OS 18.8 months), UPS (median OS 7.0 months) and AS (median OS 5.4 months).

### Toxicity

Twenty-four patients (33.3%) experienced at least one AE. In total, 36 AEs (all grades) were observed, including five grade 3–4 AEs in five patients. We did not identify any new safety signal. Seven patients (9.7%) required a delay of treatment due to toxicity (adrenal insufficiency grade 2, suspected immune-mediated encephalitis grade 3, oral mucositis grade 2, colitis grade 3, memory impairment grade 3 and diarrhea grade 2). The 7th patient developed Cushing’s syndrome due to exposure to glucocorticoids during radiation therapy for spinal cord compression from bone metastases. In six patients (8.3%), there was any grade AE which led to therapy permanent discontinuation (suspected immune-mediated encephalitis grade 3, suspected cellulitis-like skin disorder grade 2, colitis grade 3, memory impairment grade 3, pneumonitis grade 3 and myositis grade 3).

## Discussion

There are challenges in developing immunotherapy for sarcomas, including the profound heterogeneity of these diseases and the immunosuppressive microenvironment of most sarcoma subtypes. A recent study demonstrated that the use of off-label ICI in sarcomas has increased over the last 10 years, especially in patients with high-grade sarcomas and those treated in academic centers [13]. A number of phase 2 prospective clinical trials in all sarcomas and more recently subtype-specific have provided efficacy and safety data. Given the small number of patients and the noncomparative design of these trials, it is difficult to draw conclusions on the clinical activity of ICI in each sarcoma subtype. ORR to ICI monotherapy has ranged between 0 and 20% in the different trials that included various subtypes [4, 5], which is low compared to other cancer types. ORR to combination ICI (PD-1/PD-L1 and CTLA4 inhibitors) is moderately higher in sarcoma patients [4, 6, 14]. Combinations of ICI with TKI (nivolumab + sunitinib, pembrolizumab + axitinib, durvalumab + pazopanib, etc.) have led to ORR >20% in unselected STS populations [7, 8, 15]. The single-arm, phase 2 study of atezolizumab in ASPS, which showed a sustained response in approximately one third of the patients and led to its approval, is a new paradigm of a very well-defined clinical trial [9].

Real-world data generated by retrospective studies are valuable tools for studying new therapies in a broad population that represents the real clinical setting. Here we present the results of a retrospective analysis of the use of ICI-based therapies in patients with advanced sarcomas across different European institutions. We report an ORR of 34.4%, which is numerically higher than in the different phase 2 trials of ICI as a further line of treatment in all-comers. This is probably due to focused patient selection, given that a significant number of our patients started on immunotherapy over the last 2 years, when correlative clinical data were available on the efficacy of ICI per sarcoma subtype and other clinical characteristics. Therefore, AS (9.7%) and the subgroup of patients with a primary tumor located in the head and neck (15.3%) were over-represented in our population. Furthermore, a large portion of our patients (38.9%) received ICI as first or second-line therapy.

There are several retrospective studies reporting on the outcomes of ICI-based regimens in sarcoma patients. A large retrospective study of 88 patients with advanced STS in four USA centers found an ORR to ICI as third-line therapy of 23.9% [16]. LMS, which is thought to be a ‘cold’ tumor, was among the subtypes with the highest ORR in that study. A smaller retrospective analysis of 40 STS patients from a single institution in the USA reported a lower ORR to double ICI of 15% [17]. Similar ORR have been reported by other retrospective analyses in USA [18]. A Chinese single-institution study of 61 STS patients reported an ORR to ICI plus TKI combination of 30% [19], which compares well with our findings. As already shown, the ORR was higher for certain subtypes, including ASPS, MFS and UPS. These histotypes, as well as AS and Kaposi sarcoma, exhibited the highest ORR in a large retrospective study from Stanford [20], suggesting that histology clearly matters. The higher ORR we observed in LMS and LPS is probably the result of the low number of patients per histotype in our study. In addition, subtype-specific retrospective analyses have also been published. According to the largest worldwide registry of ASPS, ORR to ICI regimens is almost 55% [21]. Other subtypes, such as dedifferentiated CS (20%) [22] and pleomorphic high-grade sarcoma (9.1%) [23], have exhibited lower ORR. Our single patient with dedifferentiated CS achieved a radiological response; in contrast the two chordoma patients did not have a radiological response.

In our study the median PFS was 4.6 months and median OS 18.8 months for the whole cohort. These outcomes are in line with different prospective phase 2 trials of ICI regimens, including the combination of durvalumab with tremelimumab [6], the combination of nivolumab with sunitinib [7] and the combination of pembrolizumab with axitinib [8], reporting a median PFS of 4.5, 5.6 and 4.7 months, respectively. In the different retrospective series, PFS varies between 2.7 [17] and 7 [24] months in unselected sarcoma populations.

Only line of therapy and response to ICI had an impact on PFS. Similarly, longer PFS has been reported in the first-line setting of advance sarcomas, with pembrolizumab in combination with doxorubicin [25] and nivolumab/ipilimumab in combination with trabectedin [26]. An association between response to ICI and survival has also been described for ASPS [21]. Furthermore, location of the primary in the head and neck and occurrence of AE showed a non-significant trend towards an improved PFS. Location of the primary tumor has already been reported to affect clinical outcome of immunotherapy, as exemplified by cutaneous primary sarcoma [18]. In another study of AS patients treated with ICI, more benefit was observed in head and neck cutaneous tumors [27]. This is probably due to a difference in immune cell composition according to the location and the size of the primary tumor [28]. The development of immune-related AEs has been associated with improved OS and a trend for improved PFS in advanced sarcoma patients treated with ICI [29]. This is also the case for other tumor types, like hepatocellular carcinoma [30]. OS was affected, except for line of therapy and response to treatment, by histology, unlike PFS. A clear relationship between outcome of ICI and sarcoma subtype has been established so far, with ASPS, UPS and cutaneous AS benefitting the most from treatment [18, 31–33].

There is a need for biomarker drive in future clinical trials. Our analysis revealed that only a small percentage of our sarcoma patients’ tumors had biomarkers predictive of response to ICI, which is in accordance with prior studies [34, 35]. The most promising biomarker for immunotherapy in sarcoma is the presence of TLS [12], as it was demonstrated in the prospective phase II PEMBROSARC trial of pembrolizumab in STS. Although specific pathology guidelines have been published for the screening of TLS through immunohistochemistry [36], it still seems challenging in clinical practice. The tumor microenvironment seems to play an important role for the response to immunotherapy. Genomic analysis of AS of the head and neck revealed a high TMB and a dominant ultraviolet damage mutational signature [37], providing a rationale for ICI use. TMB has been associated with clinical outcome of ICI in cutaneous AS [27]. Recently, sarcoma ‘ecotype’, designated by cellular communities and fundamental transcriptionally-defined cell states through artificial intelligence, was found to correlate with prognosis in patients treated with immunotherapy [38], but this finding needs validation in prospective clinical trials.

We acknowledge that our study has certain limitations. The unselected study population, coupled with the relatively small numbers of patients per subtype, limits the efficacy analysis. Furthermore, our patients were treated with different ICI-based regimens (monotherapy and combinations), rendering the interpretation of the results more complex. Due to the retrospective design, there is a non-rigid patient selection, the disease assessment intervals were not fixed and there is a heterogeneity in assessing best response to treatment and toxicities. Furthermore, central pathology review was not required for inclusion in this study, although all pathology was reviewed by experienced sarcoma pathologists.

Future strategies of immunotherapy in sarcomas focus on novel drug combinations and alternative approaches, such as adoptive cell therapy. New and probably more effective ICIs are being developed, such as the combination of botensilimab + balstilimab [39]. Numerous combinations of ICI with other drugs are currently being investigated, including nivolumab +/- pazopanib (NCT03149120), pazopanib +/- pembrolizumab (NCT05679921), nivolumab + relatlimab (NCT04095208) and nivolumab + chemotherapy (NCT04339738) in advanced STS. Subtype-tailored approaches are gaining ground, such as the combination of palbociclib and retifanlimab (an anti-PD1 inhibitor) in DDLPS (NCT04438824). In addition, ICI are likely to be used in the neoadjuvant/adjuvant setting, according to relevant clinical trials; neoadjuvant nivolumab +/- ipilimumab has been proved efficacious in UPS [40], whereas pembrolizumab was tested in combination with radiation therapy as neoadjuvant/adjuvant treatment in high-risk UPS and LPS of the extremity (NCT03092323). Optimal patient selection for immunotherapy will be based on histology and tumor biology.

## Supplementary Material



## Data Availability

The data presented in this study are available on request from the corresponding author.
