# Supplementary material for: Real-world data on immune checkpoint inhibitors in advanced sarcomas across multiple European institutions
Source: Acta Oncol. 2025 Jun 10;64:43135. doi: 10.2340/1651-226X.2025.43135 (PMC12171866; doi:10.2340/1651-226X.2025.43135)

Supplementary material has been published as submitted. It has not been copyedited, or typeset by Acta Oncologica

Supplementary Figure 1. Progression-free survival stratified by line of treatment of immune checkpoint inhibitors.

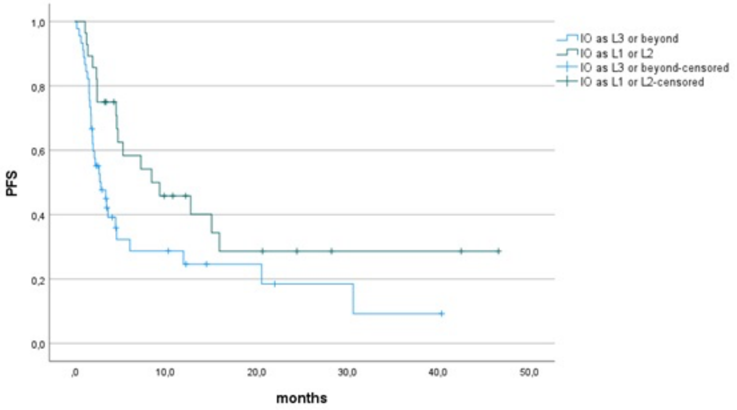

Supplementary Figure 2. Progression-free survival stratified by best response to immune checkpoint inhibitors.

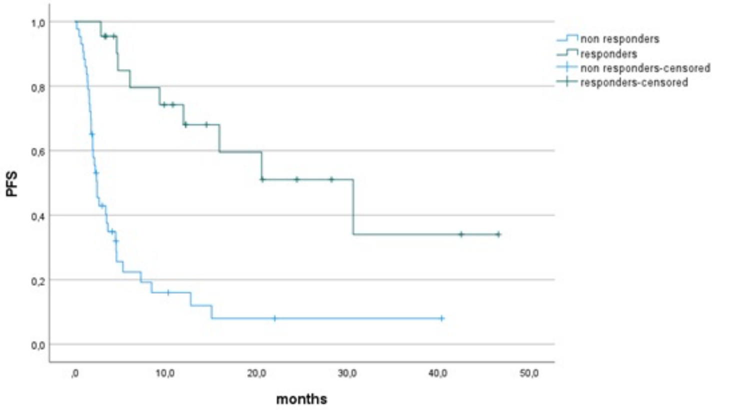

Supplementary Figure 3. Overall survival stratified by line of treatment of immune checkpoint inhibitors.

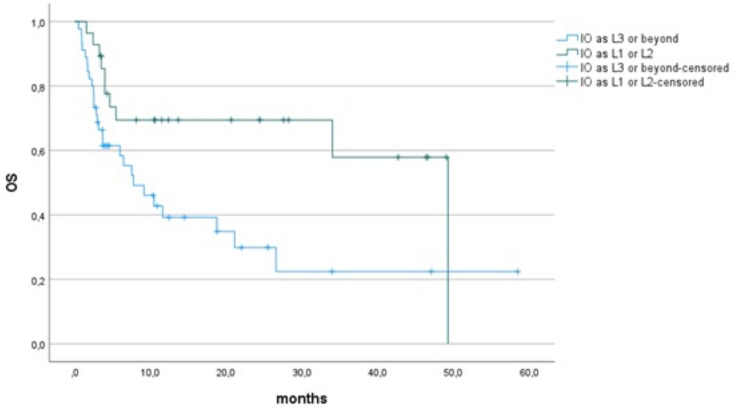

Supplementary Figure 4. Overall survival stratified by best response to immune checkpoint inhibitors.

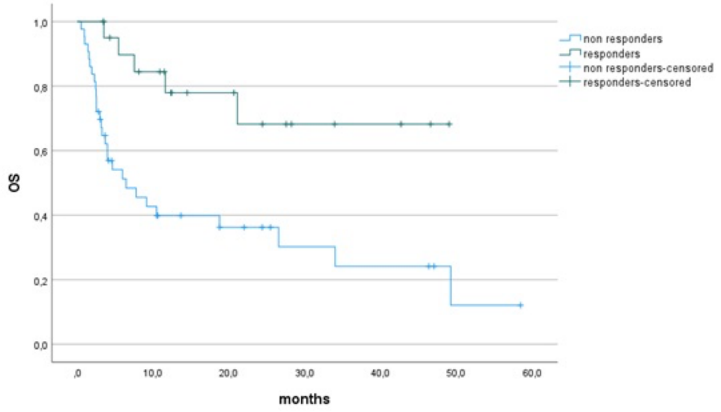

Supplement: Supplementary file 1 [file AO-64-43135-s1.pdf]
